# Examination of the immunohistochemical localization and gene expression by RT-PCR of the oxytocin receptor in diabetic and non-diabetic mouse testis

**DOI:** 10.22038/IJBMS.2018.28069.6820

**Published:** 2018-07

**Authors:** Ayşe AYDOĞAN, Seyit Ali BİNGÖL

**Affiliations:** 1 Veterinary Faculty, Histology and Embryology Department, Kafkas University, 36100, Kars, Turkey; 2 Medicine Faculty, Histology and Embryology Department, Kafkas University, 36100, Kars, Turkey

**Keywords:** Diabetes, Immunohistochemistry Oxytocin receptor, RT-PCR Testis

## Abstract

**Objective(s):**

The aim of this study was to determine Oxytocin receptor (OTR) gene expression and localization in diabetic and non-diabetic mouse testes by RT-PCR and immunohistochemistry, respectively.

**Materials and Methods:**

In this study, 18 male BALB/c mice (8–12 weeks old) were used and divided into three groups: diabetic, sham, and control. Streptozotocin (STZ) was applied to the diabetic group and sodium citrate was administered to the sham group in the same way, however, the control group was left untouched. The testicular tissues were removed on the thirtieth day of testing; the right testis tissues were passed through a routine histologic process and sections were stained with H&E and PAS staining techniques. The avidin-biotin-peroxidase method was applied to determine OTR immunoreactivity, while the left testis tissues were used for RT-PCR.

**Results:**

It was found that the body weight had decreased in the diabetic group and the diameter of the seminiferous tubules in the said group was shorter than those of the other groups. There were no obvious differences with regard to the histologic appearance between the groups. The immunohistochemical examination showed that the OTR immunoreactivity was strong in the control and sham groups but weak in the diabetic group, and the immunoreactivity was only seen in the Leydig cells. In addition, the OTR gene expression was lower in the diabetic group than in the other groups.

**Conclusion:**

We concluded that diabetes reduces the OTR expression in the testis. It is suggested that OTR protection should be researched in diabetes for healthy reproduction and sexuality.

## Introduction

An oxytocin receptor (OTR) is a molecule that keeps oxytocin (OT) on the cell membrane. Hence, OT can show its effects. Although it is known that OT has many functions in both males and females, the functions in males are not entirely known. Therefore, the OTR discovery is important to better understand the molecular mechanisms of OT (1). 

For a long time, OT was recognized as a female reproductive hormone, so its role is familiar in the gender in question. In a recent study, OT has a direct action on spermatogenesis and steroidogenesis. This means that OT also has an effect on the male reproductive system (2). It is clear now that OT is related to both reproductive organs and sexual behavior in males (3). It has three important functions in the testis; firstly, to contract the seminiferous tubules, the second role is to regulate spermatogenesis, and the third is to increase the sperm count. OT increases sperm motility and its secretion begins to rise during spermatogenesis, sexual stimulation, and arousal, and it reaches its peak during orgasm in both males and females (4). 

Diabetes mellitus is a metabolic disease characterized by hyperglycemia which is caused by a deficiency of insulin or disorders of the insulin’s actions (5). The high level of blood glucose causes the destruction of nerves and blood vessels, hence the fact that complications caused by the disease are seen in the relevant organs. Diabetes has been associated with many systemic disorders, including the reproductive system in both males and females. When its disorders cause vaginal infections and dryness in female, they cause infertility and penile erectile dysfunction in males. The sexual reluctance is one of the complications of diabetes in both genders (6, 7). Furthermore, the high level of blood glucose increases the number of immature sperm, however, it decreases the number of sperm. This means that there is an inverse relationship between the level of blood glucose and testicular function (8). 

The aim of the present study was to compare OTR expression and localization in the testes of healthy and diabetic mice by RT-PCR and immunohistochemistry, respectively.

## Materials and Methods

***Experimental animals***

This research was approved by the Local Ethical Committee for Animal Experiments at Kafkas University (KAU-HADYEK-2015-041). The specimens were obtained from the Medical Experimental Application and Research Center at Ataturk University. For this study, 18 male BALB/c mice (8–12 weeks old) were used. The mice were kept in the Animal Experimental Application and Research Center at Kafkas University during the experiment and were fed a commercial pellet diet and tap water *ad libitum*.

Two weeks after the specimens were obtained, (which was an adaptation period for them), they were divided into three groups: sham, control, and diabetic. Streptozotocin (STZ; Sigma, St.Louis, MO) was dissolved in 0.1 M sodium citrate buffer (pH 4.5). Diabetes was induced in the diabetic group by way of a single intraperitoneal (IP) injection of STZ-Na-citrate solution at a dose of 100 mg/kg (9). Citrate buffer solution without STZ was administrated into the sham group, and the control group was left untreated. The day of the STZ injections was designated as day 0 and the experimental process took 30 days. On days 0 and 30 of the study, the blood glucose levels were measured and the mice were weighed after fasting for 12 hr.

***Determination of blood glucose level***

Blood samples were taken from the tail veins of all specimens on the first and thirtieth days. Blood glucose levels were measured by use of a glucometer (Rocho Accucheck Go) after 12 hr fasting and before the STZ injection. On the third day after the STZ injection, the diabetic group was not fed for 12 hr and the blood glucose levels were measured by the same method. The mice that were abstained from food had blood glucose levels equal to or greater than 200 mg/dl and were therefore considered diabetic (10).

***Taking tissue samples***

Tissues were taken on the thirtieth day after the STZ administration. The animals were deprived of food for 12 hr and then the final body weight of the animals and also the blood glucose levels were determined. Next, the procedure of cervical dislocation was performed under ether anesthesia, and testicular tissues of the subjects were then obtained. Additionally, both testes were weighed separately. Subsequently, the right testis was used for routine histological and immunohistochemical procedures. The left testis was homogenized in 1 ml of TRI Reagent by using a homogenizer (Wiggen Hauser) for RT-PCR analysis. 

***Histological and immunohistochemical procedures***

The right testicular tissues were fixed in 10% formalin for histological and immunohistochemical examinations. After routine histological procedures, tissues were embedded in paraffin. The sections were taken at 5 µm thickness from paraffin-embedded tissue blocks. The sections were then stained with Hematoxylin-Eosin (H&E) and Periodic Acid Schiff (PAS) staining methods to examine the histological structure of the testicular tissues. In each subject, 20 transverse sections of the seminiferous tubules with the round counter were selected randomly to measure the tubule diameter. The ocular micrometer was used to measure the diameter of the seminiferous tubules.

The Avidin-Biotin-Peroxidase Complex (ABC) technique was applied to examine the OTR immunoreactivity in the testicular tissues. After the sections were deparaffinized and rehydrated, they were washed with phosphate buffered saline (PBS) (0.1 M, pH 7.2) and then incubated in 3% H_2_O_2 _for 10 min at room temperature to block endogenous peroxidase activity. After a second wash with PBS for antigen retrieval, the sections in 0.1 M citrate buffer (pH 6.0) were heated in a microwave oven at 600 watts for 10 min. The sections were washed again with PBS and then they were incubated in UV serum (%10) (Ultra V Block, Fremont, CA) for ten minutes to block non-specific binding. After washing with PBS for a third time, the sections were incubated with the anti-OTR antibody (ab217212, Abcam, Cambridge, MA) (1:250 dilution ratio) for 1 hr at room temperature. After the incubation with the primer antibody, the sections were washed with PBS another time and then they were incubated with the biotinylated secondary antibody (Ultravision, biotinylated goat anti-rabbit, Lab Vision, Fremont, CA), which was produced against the species in which anti-OTR was produced, for 30 min at room temperature. The sections were washed with PBS once more, before being incubated with streptavidin peroxidase (Lab Vision, CA) for 30 min at room temperature. After a final wash with PBS, the Diaminobenzidine (DAB) (Thermo TA 125-HD) technique was applied for visualization (11). Mayer’s hematoxylin was used as a nuclear counterstain.

***Gene expression***

The left testis tissue samples that were taken for gene expression were homogenized in 1 ml of TRI reagent (SIGMA, 93289) by using a homogenizer (9). Total RNA was obtained from these samples. The amount of RNA in 1 µl was measured with a spectrophotometer at a wavelength of 260 nm and then a solution containing 3 µg RNA was calculated. For each sample, 1 µl of oligo dT primers were added to the solution containing 3 µg RNA and the volume was increased to 12 µl with nuclease-free water. 

The Fermentas Revert Aid First Strand cDNA Synthesis Kit (K1622) was used for the RT reaction. All steps were performed according to the kit procedure. 4 µl of 5X reaction buffer, 1 µl RNase inhibitor, 2 µl of 10 mM dNTP mix (dATP, dCTP, dGTP ve dTTP), and 1 µl revert Aid M-MuLV reverse transcriptase enzyme were added to the samples that were obtained from the previous step. 20 µl of the mixture were kept at 42 ^°^C for 60 min and at 70 ^°^C for 5 min. The cDNA molecules obtained at the end of the program were stored at -20^ °^C. 

1 µl from samples containing cDNA, 2.5 µl 10X buffer, 2,5 µl MgCI_2_, 2 µl dNTP, 2,5 µl OTR-primer-F (5’TTC TTC GTG CAG ATG TGG AG’3), 2,5 µl OTR-primer-R (5’AGG ACG AAG GTG GAG GAG TT’3) (12), 0,5 µl Taq DNA Polymerase, and 11.5 ddH2O were put into each tube and the tubes were then placed in a thermal cycler. A program was performed at 94 ^°^C for 2 min, followed by 40 cycles (at 94 ^°^C for 1 min, at 60 ^°^C for 1 min, and at 72 ^°^C for 1 min), ending with 72 ^°^C for 5 min.

After amplified DNA was obtained, the last samples of DNA were kept at -20 °C. The same steps were applied to the control gene (β-actin-primer-F, 5’TCA G AA TGT ACG TGA ATC GT’3, β-actin-primer-R, CCT GAA CAT TGC GTG CAC GATG’3). 

For the DNA to be visible under UV, 12.5 µl of ethidium bromide was added to 1.5% agarose gel. Afterward, the gel with ethidium bromide was put in a gel tray and a comb was inserted into the gel to create the wells. The gel was left to form a mold at room temperature for 45 min and then the comb was removed and the gel mold was placed in the gel box.

For each subject, 10 µl from the last sample and 2.5 µl of loading dye (G7654) were put in a tube. The leader (10 µl) was put in the first and last wells and each sample along with the loading dye was put into other wells. The DNA in samples ran at 90 V for 50 min and the amplified products were looked at and photographed under a UV light. The expression levels of OTR were measured densitometrically and evaluated statistically.

***Statistical analysis***

SPSS 16.0 for Windows was used for statistical analysis. One-way analysis of variance (ANOVA) test was used for multiple comparison analysis. Bonferroni or Tamhane was used according to the results of a homogeneity variance test. Student’s t-tests were used for the comparison of two means and the differences between these groups were considered to be significant at *P*<0.05.

## Results

***Live weight***

The body weights of the specimens within each group were determined on the first and the thirtieth days of the study. The results revealed very little difference in weight between these two days within the control and the sham groups. In contrast, there was a significant decrease in weight on day thirty compared with day one in the diabetic group (*P*<0.05) (Table 1).

**Table 1:** The live weights were compared within each group according to the days.

Groups and days	N	Live weight (g)±SD	*P*-value
Diabetic 0^th^ day	6	37.00 ± 4.5	0.048*
Diabetic 30^th^ day	6	31.66 ± 2.8	
Sham 0^th^ day	6	33.33 ± 4.2	0.136
Sham 30^th^ day	6	35.83 ± 3.3	
Control 0^th^ day	6	38.66 ± 2.4	0.589
Control 30^th^ day	6	40.00 ± 3.5	

* *P*<0.05 means significant difference (SD: standard deviation). Each group was evaluated within itself separately according to the days.

**Table 2:** The comparison of testicular weights between groups

Group	N	The weight of the right testis (g) mean± SD	The weight of the left testis (g) mean± SD
Diabetic	6	0.113 ± 0.022	0.121 ± 0.012
Sham	6	0.121 ± 0.026	0.127 ± 0.011
Control	6	0.123 ± 0.013	0.128 ± 0.010
		*P*-value=0.607	*P*-value=0.867

There were no significant differences (*P*>0.05) between mean weights of the testes in each column (each column was evaluated within itself separately) (SD: standard deviation).

**Figure 1:**
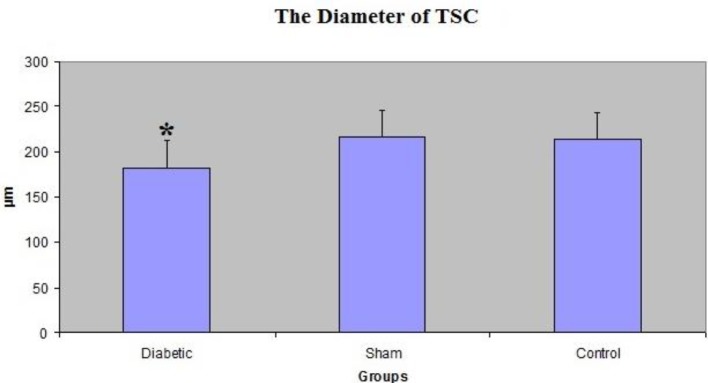
The comparison of the diameters of tubulus seminiferous contortus (TSC) between groups. The average diameter in the diabetic group of BALB/c mice was smaller than those of the others (*P<0.05)

**Figure 2:**
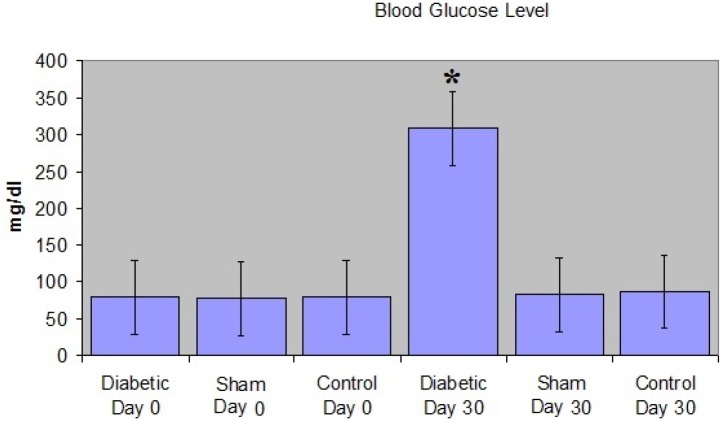
The comparison of the blood glucose levels between groups of BALB/c mice. The glucose level in the diabetic group on the 30th day was higher than the glucose levels in the sham and control groups on the 30th day and the glucose levels in all groups on the 0th day. *P<0.05

**Figure 3:**
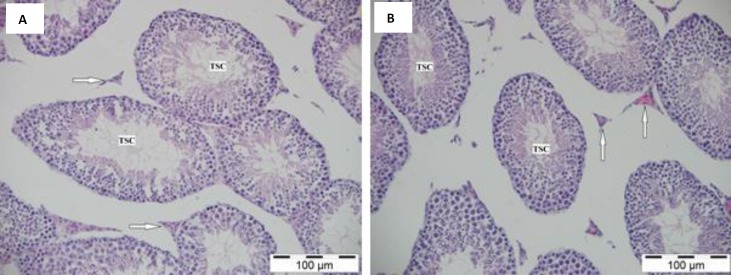
The normal histologic appearance of the testis in the control (A) and diabetic (B) groups. H&E stain. Arrow: Interstitial area. TSC: Tubulus seminiferous contortus. Bar: 100 μm

**Figure 4:**
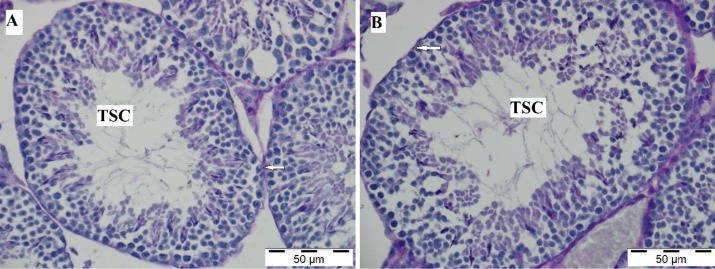
The histological structure of testis in the control (A) and diabetic (B) groups. The basal membrane surrounded the tubulus seminiferus contortus. PAS stain. Arrow: Basal membrane of tubulus seminiferous contortus (TSC). Bar: 50 μm

**Figure 5:**
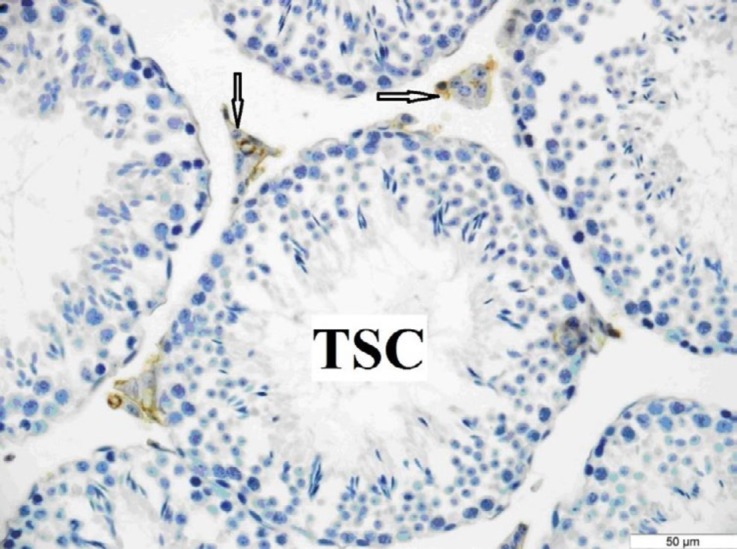
OTR immunoreactivity in the testis of the control group. OTR immunoreactivity was seen only in the interstitial area. Arrows: Interstitial area. TSC: Tubulus seminiferous contortus. Bar: 50 μm

**Figure 6:**
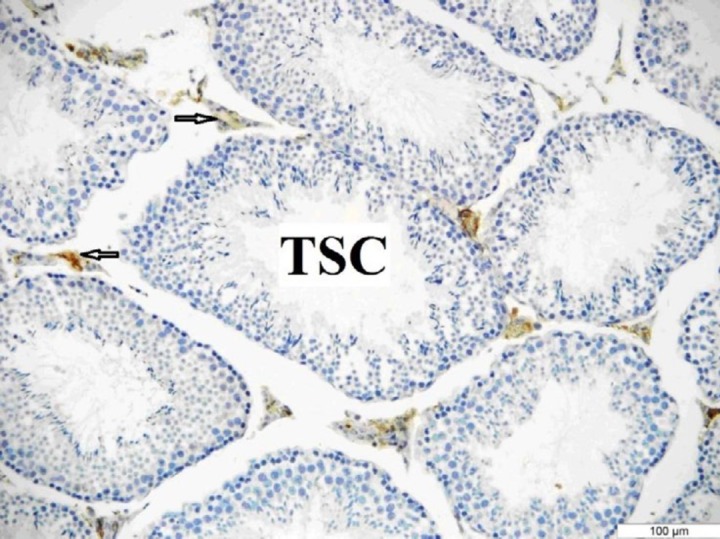
OTR immunoreactivity in the testis of the sham group. The mice were injected with sodium citrate. OTR immunoreactivity was seen only in the interstitial area. Arrow: Interstitial area. Bar: 100 μm

**Figure 7:**
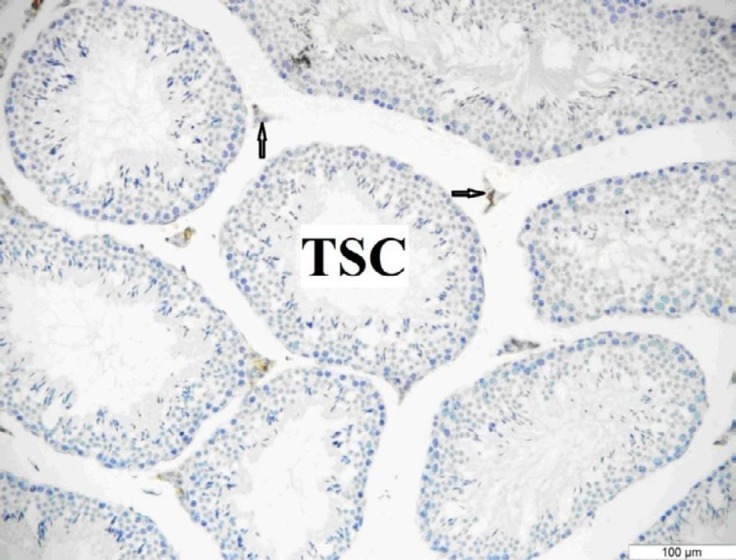
OTR immunoreactivity in the testis of the diabetic group. The mice were injected with STZ (100 mg/kg, IP). There was weak OTR immunoreactivity only in the interstitial area. Arrow: Interstitial area. Bar: 100 μm

**Figure 8:**
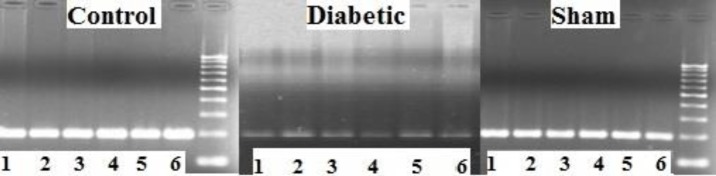
The view of PCR gel for OTR gene in the testes of control, diabetic, and sham groups of BALB/c mice

**Figure 9:**
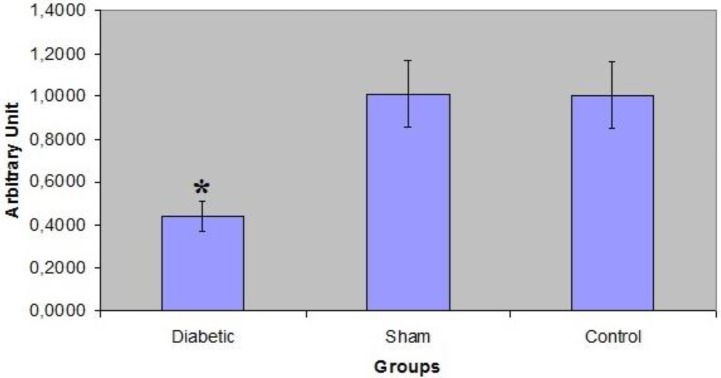
OTR Gene expression level in the testes. OTR expression in the testes of the diabetic group of BALB/c mice was lower than in the sham and control groups. *P<0.05

***Testicular weight***

The weights of the left testes of groups were compared to each other and the same procedure was also conducted on the right testes. There were no statistically notable differences between these groups in terms of either the right or left testicular weights (*P*>0.05) (Table 2).

***Histometry of tubulus seminiferous contortus***

Statistically, there were differences between the tubule diameters of these groups (*P*<0.05). For instance, the average diameter in the diabetic group (182.28 ± 6.7 µm) was smaller than those of the others (sham: 215.76 ± 11.3 µm, control: 213.90 ± 11.4 µm) (Figure 1). 

***The blood glucose level***

The blood glucose levels of the three groups obtained on 0^th^ and 30^th^ days were compared statistically. It was found that the glucose levels of all these groups on the 0^th^ day and those of the sham and control groups on the 30^th^ day were very similar to each other. However, the glucose levels in all groups were notably lower than that of the diabetic group (309.00±53.5 mg/dl) on the 30^th^ day (Figure 2). 

***Histology***

The histologic examination of the testis showed that the tubulus seminiferous contortus, spermatogenic cells, and interstitial areas in the testis had a normal histologic appearance in both the control and sham groups (Figure 3A). In the diabetic group, spermatogenic cells, Leydig cells, and interstitial areas had similar appearances to those of the other groups (Figure 3B). It was observed that a thin layer of the basal membrane completely surrounded the tubulus seminiferous contortus. It was PAS positive and its appearance was similar in all groups (Figures 4A and B).

***Immunohistochemistry***

The OTR immunoreactivity was more prominent in the testes of the control and sham groups than in the diabetic group. It was determined that there was OTR immunoreactivity only in the Leydig cells in all groups (Figure 5, 6, 7).

***Gene expression***

It was determined that there was a noteworthy difference between the groups in terms of OTR expression. According to the RT-PCR results, OTR expression in the diabetic group was much lower than in the sham and control groups (Figures 8 and 9).

## Discussion

In this study, OTR localization and expression in the testis, live weight, testicular weight, and testicular histology were investigated in the diabetic (diabetes induced by STZ) and healthy mice. 

Researchers reported that the live weights in diabetic mice decreased on either the 20^th^ or 30^th^ day (13). In a study it was detected that the live weight gradually reduced in the diabetic group during a 24-week observation (14). Other researchers mentioned that the diabetic rats lost weight (15). There was an important difference between the diabetic and control groups regarding live weight in another study (16). Orman *et al.* noted a significant reduction in live weights of diabetic mice (17). According to one study diabetes did not cause a decrease in the live weights of mice (18). In our study, we found a remarkable reduction in the live weight of the diabetic group on day 30.

It was reported that testicular weight decreased in the diabetic mice like live weight (19). In the present study, although the right and left testicular weights of the diabetic group were lighter than those of other groups, there were no significant differences between the means of the groups in terms of the testicular weight.

In one study there was a reduction in the spermatogenic cells, atrophy, and disruption in the seminiferous tubules of the diabetic rats (20). Guneli *et al.* Akkoc *et al.,* and Orman *et al.* determined that diabetes caused the basal membrane of the seminiferous tubules to become thicker (17, 21, 22). Orman *et al.* notified that germ cells with large nuclei, vacuolization, and atrophy were seen in the seminiferous tubules of the diabetic group (17). In the current study, no evident histological difference was observed between the groups.

Guneli *et al.* informed that the diameters of the seminiferous tubules in the diabetic group were smaller than those of the control group (21). Orman *et al.*, Akkoc *et al.,* and others notified that diabetes was a reason for decreased diameter of the seminiferous tubules (17, 22, 23). In the current study, there was a significant difference between the groups with regards to the diameter of the seminiferous tubules. There was a vast decrease in the diameter of the seminiferous tubules (182.28 ± 6.7 µm) in the diabetic group compared to those of the sham (215.76 ± 11.3 µm) and control (213.90 ± 11.4 µm) groups.

A study examined the effect of OT on testicular tissue in STZ-induced diabetic rats and reported that OT played an effective role in diabetes (24). Anti-inflammatory effect of OT was indicated (25) and researchers informed that OT had a role in male and female reproductive behavior (26). It was noted that intranasal OT administration had a decreasing effect on testosterone in healthy men (27) and presence of OT was mentioned in the Leydig cells in several studies of mammals (28, 29). OT induced penile erection and yawning according to a study (30). All of the effects of OT can only occur with the presence of OTR.

OTR immunoreactivity in the rat penis was seen in endothelial cells and smooth muscle cells around the artery (31). Another study mentioned that OTR was present in stromal cells in human prostatic tissue (28). It was noted that OTR immunoreactivity was present in smooth muscle cells of the monkey epididymis (28, 29). Also, although OT was only present during spermatogenesis, OTR was seen in Leydig cells before spermatogenesis and even before puberty (29). OTR immunoreactivity was seen in both Leydig cells and Sertoli cells of humans. In the same study, it was told that OTR immunoreactivity was seen in the Leydig cells of monkeys (28). OTR immunoreactivity was observed in both Leydig cells and Sertoli cells in a research (32). In our study, OTR immunoreactivity was observed only in the Leydig cells but not in the seminiferous tubules.

It was mentioned that OTR expression in a rat’s penis increased in the diabetic group compared to the control group (31). The effect of OTR on penile erection was examined in rats by RT-PCR and it was stated that OTR affected penile erection in different forms at different expression levels (33). The expression of the OTR gene was observed in the testis and epididymis, and OTR expression in the cryptorchid group was less than that of the normal group (34). According to the findings of the molecular analysis of our study, a considerable decrease in the OTR expression was observed in the diabetic group compared to the control and sham groups.

## Conclusion

OT is important for sustaining a healthy sexual function in both genders. These functions only depend on the cell membrane OTR. We concluded that diabetes has a negative effect on OT in the testis by reducing the OTR expression. OTR protection may be suggested in diabetes for healthy reproduction and sexuality. 
